# Defective proteostasis in induced pluripotent stem cell models of frontotemporal lobar degeneration

**DOI:** 10.1038/s41398-022-02274-5

**Published:** 2022-12-10

**Authors:** Sidhartha Mahali, Rita Martinez, Melvin King, Anthony Verbeck, Oscar Harari, Bruno A. Benitez, Kanta Horie, Chihiro Sato, Sally Temple, Celeste M. Karch

**Affiliations:** 1 Department of Psychiatry, Washington University in St Louis, St Louis, MO USA; 2 Department of Neurology, Washington University in St Louis, St Louis, MO USA; 3 Hope Center for Neurological Disorders, Washington University in St Louis, St Louis, MO USA; 4 Neural Stem Cell Institute, Rensselaer, NY USA

**Keywords:** Personalized medicine, Stem cells, Molecular neuroscience

## Abstract

Impaired proteostasis is associated with normal aging and is accelerated in neurodegeneration. This impairment may lead to the accumulation of protein, which can be toxic to cells and tissue. In a subset of frontotemporal lobar degeneration with tau pathology (FTLD-tau) cases, pathogenic mutations in the microtubule-associated protein tau (*MAPT*) gene are sufficient to cause tau accumulation and neurodegeneration. However, the pathogenic events triggered by the expression of the mutant tau protein remain poorly understood. Here, we show that molecular networks associated with lysosomal biogenesis and autophagic function are disrupted in brains from FTLD-tau patients carrying a *MAPT* p.R406W mutation. We then used human induced pluripotent stem cell (iPSC)-derived neurons and 3D cerebral organoids from patients carrying the *MAPT* p.R406W mutation and CRISPR/Cas9, corrected controls to evaluate proteostasis. *MAPT* p.R406W was sufficient to induce morphological and functional deficits in the lysosomal pathway in iPSC-neurons. These phenotypes were reversed upon correction of the mutant allele with CRISPR/Cas9. Treatment with mTOR inhibitors led to tau degradation specifically in *MAPT* p.R406W neurons. Together, our findings suggest that *MAPT* p.R406W is sufficient to cause impaired lysosomal function, which may contribute to disease pathogenesis and serve as a cellular phenotype for drug screening.

## Introduction

Frontotemporal lobar degeneration with tau pathology (FTLD-tau) is characterized clinically by behavioral abnormalities along with memory loss and neuropathologically by the accumulation of intracellular tau protein. A subset of FTLD-tau cases occur by familial mechanisms in which mutations in the *MAPT* gene, which encodes the tau protein, are dominantly inherited.

Several mechanisms have been proposed to explain how the more than 50 reported *MAPT* mutations cause disease: abnormal *MAPT* splicing, altered microtubule binding kinetics, impaired degradation, or tau accumulation and aggregation, among others [1]. All *MAPT* mutations share an increased propensity for the tau protein to accumulate [2]. One such mutation, *MAPT* p.R406W, presents with Alzheimer disease-like progressive memory decline and exhibits a protracted clinical course that may last decades [3–5]. How *MAPT* p.R406W mutations lead to tau aggregation and precipitate their characteristic neurodegenerative changes is not clear.

Despite an incomplete picture of how the *MAPT* p.R406W mutation leads to disease, increasing evidence implicates impairment of the neuronal endolysosomal pathway in FTLD-tau [6–13]. However, how neurons expressing mutant tau degrade tau and whether mutant tau is sufficient to drive disrupted protein homeostasis (e.g., proteostasis) remains poorly understood.

Patient-derived induced pluripotent stem cells (iPSC) have emerged as a powerful tool to study the molecular mechanisms underlying neurodegenerative diseases [14–17]. iPSCs have the potential to more faithfully reflect the endogenous expression and splicing of genes (e.g., *MAPT)* compared to models that rely on overexpression, giving us a path towards understanding the factors that contribute to disease [18, 19]. To date, iPSC carrying *MAPT* mutations p.N279K, p.P301L, p.V337M and IVS10 + 16 have been described, and display phenotypes such as tau accumulation, tau hyperphosphorylation, tau insolubility, and vulnerability to specific cellular stressors [20–36]. In iPSC-neurons from *MAPT* p.R406W carriers, mutant neurons exhibit altered pre-synaptic function, tau mislocalization, altered tau phosphorylation and fragmentation [19, 35, 36].

Here, we show that iPSC-neurons from *MAPT* p.R406W carriers exhibit morphological and functional deficits in the lysosomal pathway that were reversed upon CRISPR/Cas9-mediated correction of the mutant allele. Additionally, we observed an increased co-localization of total and phosphorylated tau with lysosomal vesicles. Thus, our findings suggest that *MAPT* p.R406W is sufficient to cause altered lysosomal function, which may contribute to disease pathogenesis.

## Materials and Methods

### Patient consent

The Washington University School of Medicine Institutional Review Board reviewed the protocol of the Knight Alzheimer Disease Research Center (ADRC) Neuropathology Core, from which clinically and neuropathologically well-characterized brain tissues were obtained. As tissue was obtained postmortem it was exempt from IRB approval. Research participants provided autopsy consent limited to removal of the brain. All data were analyzed anonymously.

Skin biopsies were performed following written informed consent from the donor. The informed consent was approved by the Washington University School of Medicine Institutional Review Board and Ethics Committee (IRB 201104178 and 201306108).

### Transcriptomics in human brain tissue

To evaluate the impact of *MAPT* p.R406W on lysosomal genes, we queried summary statistics from our previously generated bulk RNAseq data of the insular cortex of *MAPT* p.R406W (*n* = 2) and neuropathology-free controls (*n* = 2) [19]. Briefly, DNA libraries of individual samples were constructed using the TruSeq Stranded Total RNA Sample Prep with Ribo-Zero Gold kit (Illumina) and then sequenced by the HiSeq 4000 (Illumina). FASTQ files were aligned to human GRCh37 primary assembly. After alignment, Salmon (v. 0.7.2) was used to quantify expression levels of individual genes included in the GENCODE reference genome (GRCh37.75). Differential gene expression was performed using the R (v.3.4.2) package DESeq2 (v.1.18.1) as previously described [19]. From the summary statistics, we extracted genes that are regulated by Transcription Factor EB (TFEB), defined as: [1] containing a Coordinated Lysosomal Expression and Regulation (CLEAR) sequence or [2] being altered by overexpression of TFEB in vitro [37]. To determine whether genes that are regulated by TFEB and altered in *MAPT* p.R406W brains were enriched in specific functional pathways, gene enrichment analysis was performed using ToppGene [38].

### iPSC generation and genome engineering

Dermal fibroblasts from *MAPT* p.R406W carriers (F11362 and F11421) were transduced with non-integrating Sendai virus carrying OCT3/4, SOX2, KLF4, and cMYC (Life Technologies) as previously described [19]. iPSCs that were heterozygous for *MAPT* p.R406W were edited to WT (F11362.1Δ1B06 and F11421.12Δ2A07) using CRISPR/Cas9 as previously reported [19, 39]. Mutation status was confirmed by Sanger sequencing (Supplemental Fig. 1). Cell lines were maintained in mTeSR medium (StemCell Technologies) on Matrigel. Cell lines were confirmed to be free of mycoplasma.

### iPSC differentiation

*MAPT* p.R406W iPSC and isogenic controls were differentiated into neural progenitor cells (NPCs) as previously described [19]. Briefly, iPSCs were dissociated with Accutase (Life Technologies). iPSCs were then plated at 65,000 cells per well in Neural Induction Media (NIM; Stem Cell Technologies) in a 96-well v-bottom plate to form neural aggregates. After 5 days, neural aggregates were plated on Poly-L-Ornithine (PLO) and laminin-coated plates to form neural rosettes. After 5 to 7 days, neural rosettes were isolated by enzymatic selection and cultured as NPCs. NPCs were cultured on PLO and laminin-coated plates and terminal differentiation was initiated with the addition of cortical maturation medium (Neurobasal-A (Life Technologies) supplemented with B27 (Gibco), BDNF (Peprotech), GDNF (Peprotech), cAMP (Sigma) and L-glutamate (Sigma)). Neural cultures were maintained for six weeks, a time at which cells are Tuj1-positive, express robust levels of tau, and exhibit robust action potentials [18, 40].

### Organoid generation

Human iPSCs were differentiated into 3D cerebral organoids as previously described [33, 41]. iPSCs were cultured as single cells in 10 µM of Y-27632 supplemented E8 media and transferred into individual wells of an AggreWell™800 plate (Stem Cell Technologies). The cells were incubated at 37 °C and 5% CO_2_ for 24 h. Organoids were then washed and transferred to an ultra-low attachment 10 cm plates in E6 medium supplemented with 2.5 μM Dorsomorphin (DM) (Tocris), 10 μM SB431542 (Tocris,) and 2.5 µM XAV-939 (Tocris). Media was replaced daily. On day 6 in suspension, media was replaced with neural medium (NM) containing Neurobasal-A (Life Technologies) supplemented with B27 supplement without vitamin A (Life Technologies), GlutaMax (Life Technologies), Anti-A (Life Technologies), 20 ng/ml FGF2 (R&D Systems) and 20 ng/ml EGF (Peprotech). Organoids were then cultured for an additional 19 days with daily medium changes in the first 10 days, and every other day for the final 9 days. To promote differentiation of the neural progenitors into neurons, FGF2 and EGF were replaced with 20 ng/ml BDNF (Peprotech) and 20 ng/ml NT3 (Peprotech) beginning at day 25. Beyond day 43, medium changes were performed every four days using only NM lacking growth factors.

### Antibodies

The following antibodies were used in this study: Tau5 (total tau; generously provided by Dr. Lester Binder), DAPI (Sigma), Alexa488 anti-rabbit (Life Technologies, Carlsbad, CA, USA), Alexa568 anti-mouse (Life Technologies, Carlsbad, CA, USA), beta III Tubulin (Promega), LAMP1 (Abcam, ab24170), AT180 (ptau-Thr231; Thermo Fisher Scientific, MN1040), EEA1 (Abcam, ab2900), Cathepsin D (generously provided by Dr. Stuart Kornfeld, Washington University in St Louis), Rab7 (Cell Signaling Technology, 9367S), TFEB (Abcam, ab270604), pTFEB (pSer122, Cell Signaling Technology, 86843S) and Ubiquitin (Cell Signaling Technology, 3933S).

### Immunoblotting

Cell lysates were extracted in lysis buffer (50 mM Tris pH 7.6, 1 mM EDTA, 150 mM NaCl, 1% TritonX-100, phosphatase and protease inhibitors (Millipore-Sigma)) and incubated on ice for 5 min. Lysates were then centrifuged at 14,000 × *g* for 10 min at 4 °C, and the resulting supernatant was saved for analysis. Total protein levels were assayed by bicinchoninic acid assay (BCA) assay (Thermo-Fisher). Standard sodium dodecyl sulfate-polyacrylamide gel electrophoresis (SDS-PAGE) was performed in 4–12% Criterion Tris-HCl gels (Bio-Rad) with 10 µg of total protein loaded in each well. Samples were boiled in Laemmli sample buffer prior to electrophoresis. Gels were transferred to polyvinylidene fluoride (PVDF) membranes, which were probed with the antibodies listed above. Antibodies were visualized with SuperSignal West Pico Chemiluminescent Substrate (Thermo) or Lumigen ECL Ultra (TMA-6) according to manufacturer’s instructions. Immunoblots were exposed on a Syngene G:Box iChemi XT Gel Documentation System and imaged using GeneSnap software according to manufacturer’s instructions. Band intensity was analyzed using GeneTools, and band intensity was expressed relative to the normalized control within each blot.

### Immunostaining

To evaluate the impact of *MAPT* p.R406W on the endolysosomal pathway, culture media was aspirated, and cells were washed and fixed with 4% paraformaldehyde (Sigma, St Louis, MO, USA). Cells were washed and permeabilized with permeabilization buffer (0.1% Triton X-100 in PBS). Cells were then blocked in 3% bovine serum albumin (BSA; Sigma, St Louis, MO, USA) and incubated with primary and secondary antibodies diluted in 1% BSA. Immunostained cells were then imaged (Nikon Eclipse 80i fluorescent microscope) and acquired by using Metamorph Molecular Devices software. Confocal images were acquired by using Zeiss LSM 880 II Airyscan FAST Confocal Microscope.

### Image quantification

Fiji, an ImageJ-based image analysis software [42], was used to quantify the distribution and size of LAMP1-positive vesicles. The extent of distribution of LAMP1-positive vesicles in each cell was determined by measuring the average distance between the nuclear membrane and LAMP1-stained vesicles present in the neurite in the axon hillock as described by Ouyang and colleagues [43]. To do this, the ‘straight line’ tool was selected to draw a straight line from the center of the nuclear membrane to the LAMP1 staining seen in the neurite in the axon hillock. The ‘measure’ tool was used to define the distance in micron. The size of the individual LAMP1-positive vesicles in the cell body was measured by using the plugin “find edges” in Fiji software to mark the edges. The diameter of the individual LAMP1-positive vesicles was then measured. In addition to measuring size, “find edges” allowed for the quantification of total LAMP1-positive vesicles.

Co-localization analysis was performed using Metamorph (Molecular Devices) software. The LAMP1-stained area was selected in the cell body of the neuron. The percent area of the red that overlapped with the green puncta area was calculated using Metamorph colocalization function. Pearson’s correlation coefficient (r) was used to quantify the association between LAMP1 and tau or tau phosphorylated at Thr231 (ptau). Values range from zero to one, where zero represents no pixels are co-localized and one represents co-localization of all pixels.

### Lysosomal activity assay

A fluorometric assay was performed to test the impact of *MAPT* p.R406W on activity of lysosomal enzymes. Cell lysates were homogenized in buffer containing 150 mM NaCl, 10 mM Tris 7.5, 1 mM DTT, and 0.2% Triton X-100. The substrate of β-glucuronidase was added to the cell lysate separately. β-glucuronidase cleavage was measured at 448 nm emission and 365 nm excitation in a fluorescence spectrophotometer using a standard curve ranging from 0.02 to 5 mM of 4-methylumbelliferone [44]. Protein quantification from three technical replicates and three biological replicates were averaged and expressed as mean ± standard error of the mean (SEM). Statistical difference was measured using an unpaired Student’s t-test.

To evaluate lysosomal acidity, six-week-old neurons were incubated for 5 min with 5 nM of LysoTracker® Red DND-99 diluted in the cortical maturation medium. Live cell images were acquired immediately [45]. Fluorescence intensity of LysoTracker Red staining in the soma was quantified by Fiji. Briefly, the fluorescence intensity of LysoTracker® in the soma of each cell was measured and then corrected for background fluorescence resulting in the Corrected Total Cell Fluorescence (CTCFs) values. CTCFs values were calculated for each cell and graph was plotted by normalizing CTCF relative to the isogenic, wild-type average value.

To evaluate proteolytic activity in lysosomal compartments, six-week-old neurons were incubated for 4 h with 10 µg/mL of DQ™ Red BSA diluted in the cortical maturation medium. Cells were washed once with HBSS solution. Live cell images were acquired immediately. Fluorescence intensity of DQ™ Red BSA staining in the soma was quantified by Fiji as described above. CTCFs values were calculated for each cell and graph was plotted by normalizing CTCF relative to the wild-type average value.

### Torin treatment of iPSC-neurons

After 6 weeks in culture, iPSC-neurons maintained in a 48-well plate were washed with DMEM/F12 and treated with 250 nM Torin or DMSO control. After 5 h of treatment, cell culture media was collected, centrifuged at 1000 rpm for 10 min at 4 °C to remove cell debris, and flash frozen. Cells were collected in PBS and pelleted.

### Immunoprecipitation and Mass Spectrometry (IP/MS) to quantify tau fragments

IP/MS for tau fragments was performed as previously described [40]. Briefly, CNBr-activated Sepharose beads (GE Healthcare 17-0430-01) were crosslinked to Tau1 (mouse monoclonal, provided by Drs. Nicholas Kanaan) and HJ8.5 antibodies (mouse monoclonal, provided by Dr. David Holtzman). Samples are spiked with 5 ng ^15^N labeled recombinant 2N4R tau (gift from Dr. Guy Lippens), and tau concentration is calculated using this internal standard.

Soluble tau was immunoprecipitated in detergent (1% NP-40), chaotropic reagent (5 mM guanidine), and protease inhibitors (Roche Complete Protease Inhibitor Cocktail). 30 µL of 50% slurry of the tau antibody beads were rotated with the solution for 90 min at room temperature. The beads were washed one time in 0.5 M guanidine and two times in 25 mM triethyl ammonium bicarbonate buffer (TEABC, Fluka 17902). The bound tau was digested on-beads with 400 ng MS grade trypsin (Promega, V5111) or AspN (Promega, V162A) for 16 h at 37 °C. Digests were loaded onto TopTip C18 (Glygen, TT2C18.96), desalted, and eluted per manufacturer’s instructions.

A 5 µL aliquot of the peptide resuspension was subjected to nano-Acquity LC and MS analysis. The nano-Acquity LC (Waters Corporation, Milford, MA) was fitted with HSS T3 75 µm x 100 mm, 1.8 µm column and a flow rate of 0.5 µL/min of a gradient of solution A and B was used to separate the peptides. Solution A was composed of 0.1% formic acid in MS-grade water and solution B was composed of 0.1% formic acid in acetonitrile. Peptides were eluted from the column with a gradient of 2% to 20% of solution B in 8 min, then 20% to 40% solution B for another 3 min before ramping up to 85% solution B in another 3 min to clean the column. The Orbitrap Fusion/Fusion Lumos was equipped with a Nanospray Flex electrospray ion source (Thermo Scientific, San Jose, CA). Peptide ions sprayed from a 10 µm SilicaTip emitter (New Objective, Woburn, Ma) into the ion–source were targeted and isolated in the quadrupole and were then fragmented by HCD and ion fragments were detected in the Orbitrap (resolution of 60,000, mass range 150–1200 m/z). Monitoring of hydrophilic peptides (SSRcalc < 9, all without leucine) for peptide profiling was performed on a HSS T3 300 µm x 100 mm, 1.8 µm column at a flow rate of 4ul/min with an elution occurring with a 2% to 12% solution B gradient and a spray operating on a 30 µm SilicaTip emitter.

**Fig. 1:**
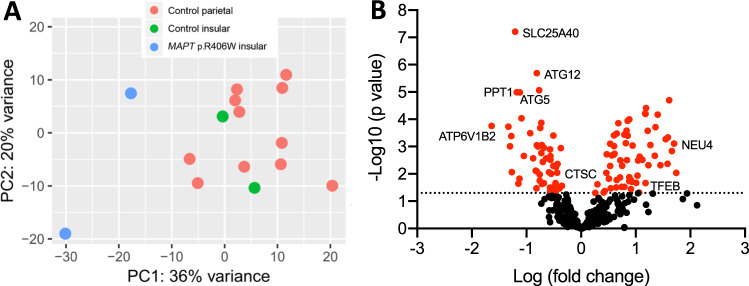
TFEB-regulated genes are differentially expressed in *MAPT* p.R406W brains. RNAseq was performed in brains from *MAPT* p.R406W and neuropathology-free controls and reported in Jiang et al. [19]. **A** Principal component analysis reveals transcriptome-wide differences between *MAPT* p.R406W (*n* = 2) and neuropathology-free control brains (*n* = 16). **B** We tested whether genes that contain a CLEAR sequence or that are differentially expressed in the presence of *TFEB* in vitro were differentially expressed in human brains from *MAPT* p.R406W carriers compared with normal controls. Volcano plot showing log2 fold change between *MAPT* p.R406W and control brains and the –log_10_ *p*-value for each gene. Red nodes: genes with *p* < 0.05.

## Results

### Lysosomal gene expression is altered in brains from *MAPT* p.R406W patients

Prior studies have demonstrated that lysosomal proteins, such as TFEB, LAMP1 and Cathepsin D, are elevated in brains from FTLD-tau patients [46, 47]. TFEB is a transcription factor that regulates lysosomal biogenesis by coordinating expression of lysosomal genes [48]. To determine whether *TFEB* gene expression is altered in FTLD-tau caused by *MAPT* p.R406W, we performed differential gene expression analysis in bulk transcriptomic data obtained from a brain region affected early in disease pathogenesis, the insular cortex, from *MAPT* p.R406W carriers (*n* = 2) and unrelated controls (*n* = 2) (Fig. 1) [19]. *TFEB* expression was significantly elevated in *MAPT* p.R406W carriers compared with unrelated controls (Table 1). TFEB regulates expression of genes containing a CLEAR-box sequence (5′-GTCACGTGAC-3) as well as some genes that lack this recognition sequence [37]. We analyzed genes with a CLEAR-box sequence (Supplemental Table 1) and genes that change as a function of TFEB overexpression (Supplemental Table 2). We found that approximately one-third of all genes regulated by TFEB are differentially expressed in brains from *MAPT* p.R406W carriers (Fig. 1, Table 1). These differentially expressed genes are enriched in pathways associated with hydrolase activity (GO:0016798; *p* = 1.15E−18) and ATPase activity (GO:0046961; *p* = 2.81E–06). TFEB has also been implicated in autophagosome formation [49]. Expression of *ATG5, ATG12*, and *ATG16L1* genes, which form a complex that is required in the early stages of autophagosome formation, were significantly lower in *MAPT* p.R406W carriers compared with unrelated controls (Table 1). *ATG2A* and *ATG2B*, which recruit other ATG proteins to the p62-positive autophagosome formation site, were also differentially expressed (Table 1) [50]. Together, these findings point to a molecular imbalance in TFEB-mediated and autophagosome pathways in *MAPT* p.R406W brains.

**Table 1:** Lysosomal genes are differentially expressed in brains from *MAPT* p.R406W carriers.

Gene	Log2 Fold Change	*p* value
*TFEB*	0.91	3.63E−02
*ABCB9*	0.43	4.24E−02
*MAN2B2*	0.47	1.92E–03
*SMPD1*	0.51	1.51E–02
*PPT2*	0.53	5.15E–03
*GAA*	0.64	3.15E–02
*ARSA*	0.65	1.68E–02
*CLCN7*	0.82	7.49E–04
*HYAL1*	0.84	3.15E–03
*GALNS*	0.88	1.00E–04
*TMEM92*	0.88	1.84E–02
*MAN2B1*	1.03	1.16E–02
*ABCA2*	1.06	4.83E–02
*HYAL2*	1.18	2.19E–02
*SGSH*	1.22	2.65E–04
*CTNS*	1.27	8.74E–04
*IDUA*	1.40	6.76E–05
*NEU4*	1.70	7.82E–04
*GGH*	−1.30	9.82E–04
*EPDR1*	−1.27	8.67E–03
*PPT1*	−1.18	1.02E–05
*ENTPD4*	−0.79	9.87E–04
*HEXB*	−0.76	1.05E–02
*PCYOX1*	−0.75	2.06E–04
*ASAH1*	−0.72	2.33E–02
*LMBRD1*	−0.70	3.66E–03
*NEU1*	−0.57	4.43E–02
*OSTM1*	−0.56	2.23E–03
*SLC17A5*	−0.51	3.94E–04
*GALC*	−0.49	2.97E–02
*CTSC*	−0.44	9.03E–03
*HPSE*	−0.42	3.00E–02
*ATG12*	−0.81	2.03E–06
*ATG5*	−1.12	1.04E–05
*ATG2A*	1.17	6.16E–05
*ATG2B*	−0.73	1.34E–04
*ATG16L1*	−0.56	3.02E–03

### *MAPT* p.R406W is sufficient to alter lysosomal protein levels in human neurons

**Fig. 2:**
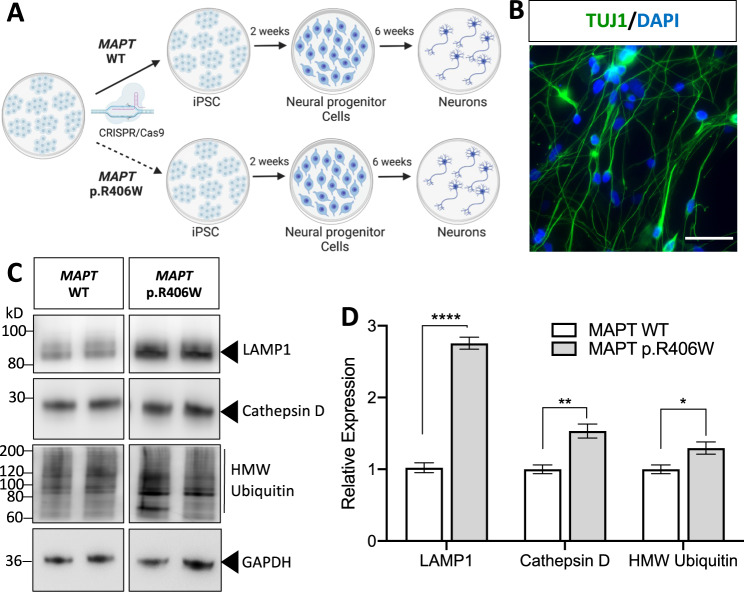
Altered proteostasis in human iPSC-neurons expressing the *MAPT* p.R406W mutation. **A** iPSC from a *MAPT* mutation carrier and CRISPR/Cas9-corrected control (wild-type (WT)) were differentiated into cortical neurons and cultured for 6 weeks prior to analysis. **B** Representative immunostaining for Tuj1 (green) and DAPI (blue) illustrates that at 6 weeks in culture, cells are enriched for neurons. Scale bar, 10 microns. **C** Immunoblots of cell lysates (10 μg total protein) were probed with LAMP1, Cathepsin D and ubiquitin antibodies. **D** Quantification of protein analyte levels in the *MAPT* p.R406W neurons and isogenic controls. Graph represents mean ± SEM. Significance was determined using an unpaired, *t*-test. *p < 0.05; **p < 0.01. ****p < 0.0001.

To determine whether *MAPT* p.R406W is sufficient to disrupt protein clearance machinery, we leveraged human iPSC isolated from *MAPT* p.R406W carriers [18, 19] (Supplemental Fig. 1). We also used CRISPR/Cas9 genome-edited, isogenic controls to evaluate whether cellular phenotypes were driven specifically by the mutant allele [18, 19] (Supplemental Fig. 1). Two independent *MAPT* p.R406W donors and their isogenic, corrected controls (*MAPT* WT) were then differentiated into cortical neurons using a growth factor-based approach (Fig. 2A; see Methods). At 6 weeks in culture, iPSC-neurons are enriched for Tuj1-positive cells (Fig. 2B), produce spontaneous action potentials, form functional synapses and display a profile of tau isoform expression that is similar to tau found in the central nervous system [19, 40]. After 6 weeks in cortical maturation medium, neurons exhibited significantly elevated total (Tau5) and ptau (pThr231; AT180; Supplemental Fig. 2). All data points were collected from multiple biological replicates across three independent differentiations from two independent *MAPT* p.R406W donor lines.

To determine whether the *MAPT* p.R406W mutation induces downstream protein degradation pathways, we measured TFEB, phospho-TFEB, Rab7, Lysosomal Associated Membrane Protein 1 (LAMP1), Cathepsin D and ubiquitinated proteins in cell lysates from *MAPT* p.R406W neurons and isogenic, control neurons. We observed a modest increase in proteins involved in lysosomal biogenesis (total TFEB, phospho-TFEB, and Rab7 [51]) in *MAPT* p.R406W neurons, however, the difference compared to isogenic, control neurons did not reach statistical significance (Supplemental Fig. 2). We found that LAMP1 and the active form of Cathepsin D were significantly elevated in the *MAPT* p.R406W neurons compared to isogenic, control neurons (Fig. 2C, D). LAMP1 is a glycoprotein that resides on lysosomal membranes and plays a role in lysosomal integrity, pH, and catabolism [52]. Cathepsin D is a lysosomal aspartyl protease [53]. Given the observation that lysosomal protein machinery was elevated in *MAPT* p.R406W neurons, we sought to determine whether there was evidence of broader cellular and proteostatic stress by measuring ubiquitinated proteins. High molecular weight ubiquitinated proteins were significantly elevated in *MAPT* p.R406W neurons compared to isogenic, control neurons (Fig. 2C, D). Thus, *MAPT* p.R406W is sufficient to produce altered expression of lysosomal and proteostatic stress in human neurons.

### *MAPT* p.R406W alters lysosomal morphology in human neurons

**Fig. 3:**
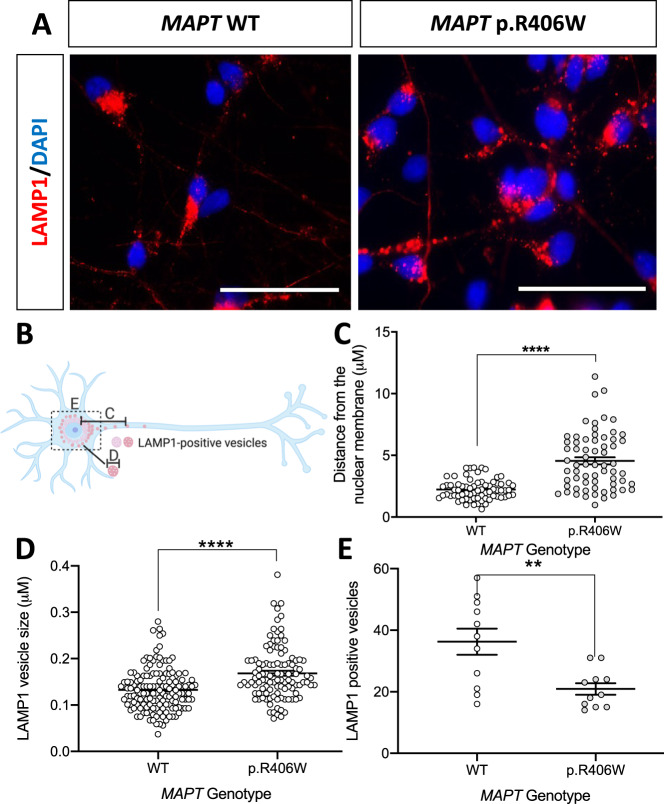
Human iPSC-neurons expressing the *MAPT* p.R406W mutation exhibit defects in lysosomal morphology. Human iPSC-neurons neurons from a *MAPT* p.R406W mutation carrier and the isogenic, CRISPR/Cas9-corrected control (wild-type (WT)) were differentiated into cortical neurons and cultured for 6 weeks prior to analysis. **A** Representative immunostaining for LAMP1 (red) and DAPI (blue) illustrates altered LAMP1-positive vesicles in the *MAPT* p.R406W neurons compared with isogenic controls. Scale bar, 10 microns. White arrows indicate LAMP1-positive vesicles in the neurites of MAPT p.R406W neurons. **B** Diagram of quantification of LAMP1-positive vesicles represented in **C**–**E**. All quantification was performed in Tuj1-positive cells. **C** Distance of LAMP1-positive vesicles from the nuclear membrane is significantly greater in *MAPT* p.R406W neurons (*n* = 27 cells) compared to isogenic controls (*n* = 60 cells). **D** The size of LAMP1-positive vesicles in the soma is significantly larger in *MAPT* p.R406W neurons (*n* = 10 cells) compared with the isogenic controls (*n* = 12 cells). **E** The number of LAMP1-positive vesicles within the soma is significantly reduced in *MAPT* p.R406W neurons (*n* = 11 cells) compared to isogenic controls (*n* = 11 cells). Graphs represent mean ± SEM. Significance was determined using an unpaired, *t*-test. *p < 0.05; **p < 0.01; ****p < 0.0001.

Alterations in lysosomal protein levels in iPSC-neurons suggests that the *MAPT* p.R406W mutation impacts lysosomal function. To begin to investigate the impact of *MAPT* p.R406W on lysosomal function, we evaluated LAMP1-positive vesicles in Tuj1-positive *MAPT* p.R406W neurons and isogenic controls. We observed a distinct pattern of LAMP1 staining between the *MAPT* p.R406W and the isogenic, control neurons (Fig. 3A). To quantify these differences, we measured three parameters (summarized in Fig. 3B): vesicle distribution (Fig. 3C), vesicle size (Fig. 3D) and vesicle number (Fig. 3E). Analyses were performed in Tuj1-positive cells (Supplemental Fig. 3).

Active lysosomes are clustered around the perinuclear region, while immature lysosomes are localized near the cell periphery [54, 55]. LAMP1-positive vesicle distribution reflects the distance of vesicles between the nuclear membrane and the cell periphery (neurite). We found that the distribution of LAMP1-positive vesicles in Tuj1-positive neurons was significantly greater in *MAPT* p.R406W neurons compared with isogenic, control neurons (Fig. 3C). LAMP1-positive vesicles in the *MAPT* p.R406W neurons were also larger in size (Fig. 3D) and fewer in number (Fig. 3E) compared to isogenic, control neurons. We replicated this finding in an independent donor carrying the *MAPT* p.R406W mutation and its corresponding isogenic control (Supplemental Fig. 4). To determine whether the *MAPT* p.R406W mutation produce a generalized defect in vesicle trafficking, we analyzed the distribution and size of early endosomes by monitoring Early Endosome Antigen 1 (EEA1)-positive vesicles in these neurons. We found no difference between the *MAPT* p.R406W and isogenic, control neurons (Supplemental Fig. 5). Thus, *MAPT* p.R406W neurons exhibit defects in lysosomal morphology.

### *MAPT* p.R406W is sufficient to alter lysosomal function in human neurons

**Fig. 4:**
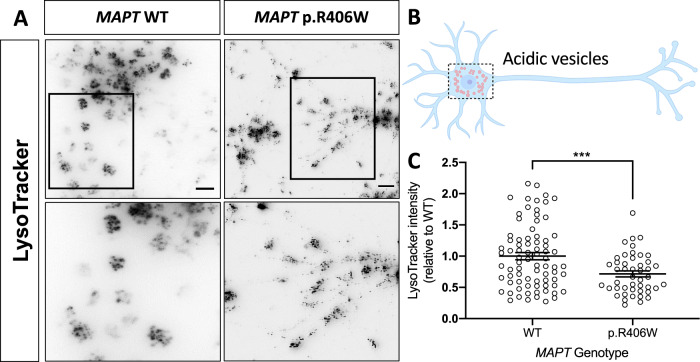
Human iPSC-neurons expressing the *MAPT* p.R406W mutation display defects in vesicle acidity. Human iPSC-neurons neurons from a *MAPT* p.R406W mutation carrier and isogenic, CRISPR/Cas9-corrected control (wild-type (WT)) were differentiated into cortical neurons and cultured for 6 weeks prior to analysis. **A** *MAPT* p.R406W neurons exhibit reduced LysoTracker staining. Live cells were incubated with Lysotracker and were imaged as described in Methods. Representative images of LysoTracker-stained neurons are shown in gray scale for clarity. Scale bar, 10 microns. Lower panel represents magnification of the cells in the black box. **B** Schematic of the quantification of LysoTracker staining in *MAPT* p.R406W neurons (*n* = 78 cells) and isogenic controls (*n* = 46 cells). **C** Quantification of the intensity of LysoTracker staining in individual soma. Graphs represent mean ± SEM. Significance was determined using an unpaired, *t*-test. ***p < 0.001.

To determine whether the observed morphological changes in LAMP1-positive vesicles in *MAPT* p.R406W neurons translates to altered lysosomal function, we evaluated vesicle acidity using LysoTracker. Immature and dysfunctional lysosomal vesicles are less acidic [56]. LysoTracker staining was diffuse in *MAPT* p.R406W neurons, while distinct, large puncta were observed in the soma in isogenic, control neurons (Fig. 4A). Upon quantification of the LysoTracker fluorescence in the soma of individual neurons (Fig. 4B), we observed a significant decrease in LysoTracker intensity in the soma of *MAPT* p.R406W neurons compared to the isogenic, control neurons (Fig. 4C). We replicated this finding in an independent donor line with its isogenic control (Supplemental Fig. 6A, B). Thus, *MAPT* p.R406W neurons have fewer LAMP1-positive vesicles (Fig. 3E), and these vesicles are less acidic than control neurons.

**Fig. 5:**
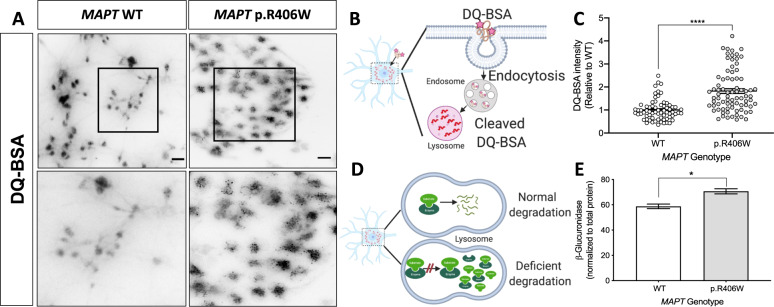
Lysosomal dysfunction in human iPSC-neurons expressing the *MAPT* p.R406W mutation. Human iPSC-neurons neurons from a *MAPT* p.R406W mutation carrier and isogenic, CRISPR/Cas9-corrected control (wild-type (WT)) were differentiated into cortical neurons and cultured for 6 weeks prior to analysis. **A** *MAPT* p.R406W neurons exhibit increased DQ-BSA fluorescence. Live cells were incubated with DQ-BSA and were imaged as described in Methods. Representative images of DQ-BSA-stained neurons are shown in gray scale for clarity. Scale bar, 10 microns. Lower panel represents magnification of the cells in the black box. **B** Diagram of DQ-BSA mechanism. **C** Quantification of the intensity of DQ-BSA staining in soma from *MAPT* p.R406W neurons (*n* = 79 cells) and isogenic controls (*n* = 69 cells). **D** Diagram of secondary elevation of lysosomal enzymes. **E** Enzyme activity of β-Glucuronidase measured in cell lysates from *MAPT* p.R406W neurons and isogenic controls. Enzymatic activity was normalized to the total protein. Graphs represent mean ± SEM. Significance was determined using an unpaired, *t*-test. *p < 0.05; ****p < 0.0001.

Next, we evaluated the impact of *MAPT* p.R406W on cargo uptake and proteolytic degradation (Fig. 5A). DQ-BSA is a fluorogenic stain that is internalized and fluoresces upon degradation by proteolytic activity in the lysosome (Fig. 5B) [57]. *MAPT* p.R406W neurons exhibited dense and punctate staining while isogenic, control neurons produced more diffuse staining (Fig. 5A). Upon quantification, we found that there is a significant increase in the DQ-BSA fluorescence intensity in the soma of *MAPT* p.R406W neurons compared to isogenic controls (Fig. 5C). We next measured the activity of a lysosomal hydrolase enzyme, β-glucuronidase, which degrades glycosaminoglycans like heparan sulfate. We observed a significant increase in β-glucuronidase activity in *MAPT* p.R406W neurons compared with isogenic controls (Figs. 5D, E). We replicated this finding in an independent donor line (Supplemental Fig. 6C, D). Together, these results suggest that *MAPT* p.R406W leads to changes in acidity and proteolysis in degradative vesicles in human neurons.

### Total tau and phosphorylated tau accumulate in lysosomes in *MAPT* p.R406W neurons

**Fig. 6:**
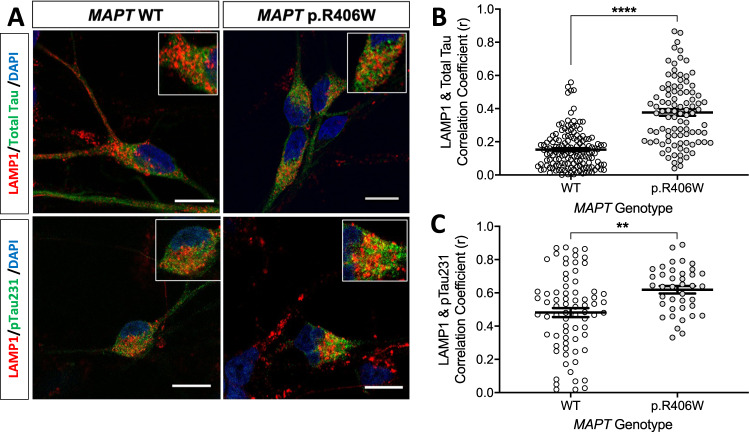
Colocalization of tau and phospho-tau with LAMP1-positive vesicles. iPSC from a *MAPT* p.R406W mutation carrier and CRISPR/Cas9-corrected control (wild-type (WT)) were differentiated into cortical neurons and cultured for 6 weeks prior to analysis. **A** Representative confocal images showing colocalization of LAMP1 (red) and total tau (Tau5; green) or ptau (AT180; green) in the *MAPT* p.R406W neurons compared with isogenic controls. Scale bar, 10 microns. **B** Colocalization of LAMP1 and total tau in tau-positive soma (WT, *n* = 146 cells; p.R406W, *n* = 95 cells) **C** Colocalization of LAMP1 and ptau in ptau-positive soma (WT, *n* = 73 cells; p.R406W, *n* = 39 cells). Graphs represent mean ± SEM. Significance was determined using an unpaired, *t*-test. **p < 0.01; ****p < 0.0001.

Tau phosphorylated at Thr231 (ptau) is enriched in tauopathy brains, particularly those carrying the *MAPT* p.R406W mutation [58–60]. We observed a significant increase in total tau and ptau by immunoblot in iPSC-neurons from *MAPT* p.R406W carriers (Supplemental Fig. 2). Specific tau post-translational modifications (e.g., phosphorylation and acetylation) have been reported to differentially clog and accumulate in lysosomes [10, 61]. Hence, we next sought to determine whether total tau and ptau accumulate in these dysfunctional lysosomes. Neurons were stained with LAMP1 and total tau (Tau5) or ptau (pThr231; AT180) and imaged by confocal microscopy. Tau-positive cells were then quantified for the extent of co-localization with LAMP1. We observed an increase in the co-localization of LAMP1 with total tau (Fig. 6A, upper panel) and LAMP1 with ptau (Fig. 6A, lower panel) in *MAPT* p.R406W neurons. Upon quantification, we detected a significant increase in the co-localization of tau (Fig. 6B) and ptau (Fig. 6C) with LAMP1-positive vesicles compared with the isogenic controls.

### *MAPT* p.R406W cerebral organoids phenocopy lysosomal alterations

**Fig. 7:**
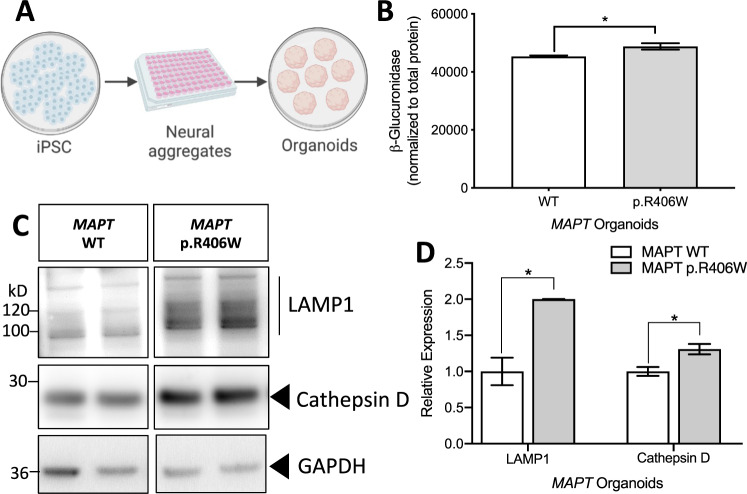
*MAPT* p.R406W organoids phenocopy lysosomal defects. **A** Schematic of organoid generation. iPSC from a *MAPT* mutation carrier and CRISPR/Cas9-corrected control (wild-type (WT)) were differentiated into cortical organoids and cultured for 2 months. **B** Enzyme activity of β-Glucuronidase measured in organoid lysates (10 μg protein) from *MAPT* p.R406W and corrected control organoids. Enzymatic activity was normalized to total protein. Graphs represent mean ± SEM. **C** Immunoblots of cell lysates from organoids were probed with LAMP1 and Cathepsin D (active form shown) antibodies. **D** Quantification of protein analyte levels in the *MAPT* p.R406W organoids and isogenic controls. Graphs represent mean ± SD. Significance was determined using an unpaired, *t-*test. *p < 0.05.

Based on the alteration in lysosomal structure and function in the *MAPT* p.R406W neurons, we hypothesized that the mutant neurons are undergoing cellular and proteostatic stress. To determine whether we could replicate our findings from the 2D, planar culture in 3D, we generated cerebral organoids from a *MAPT* p.R406W carrier and isogenic control. Culturing neurons in 3D facilitates self-organization, layering similar to what is observed in the developing human cortex, and formation of complex synaptic connections [33, 41, 62]. Following previously published protocols [41], iPSCs were patterned into neural aggregates and then cultured in suspension for 2 months prior to analysis (Fig. 7A). Similar to the 2D cultures, we observed a significant increase in β-glucuronidase activity in the *MAPT* p.R406W organoids compared to the isogenic controls (Fig. 7B). We also found that LAMP1 and Cathepsin D were significantly increased in the *MAPT* p.R406W organoids compared to the isogenic controls (Fig. 7C, D).

### mTOR inhibition reduces tau in *MAPT* p.R406W neurons

**Fig. 8:**
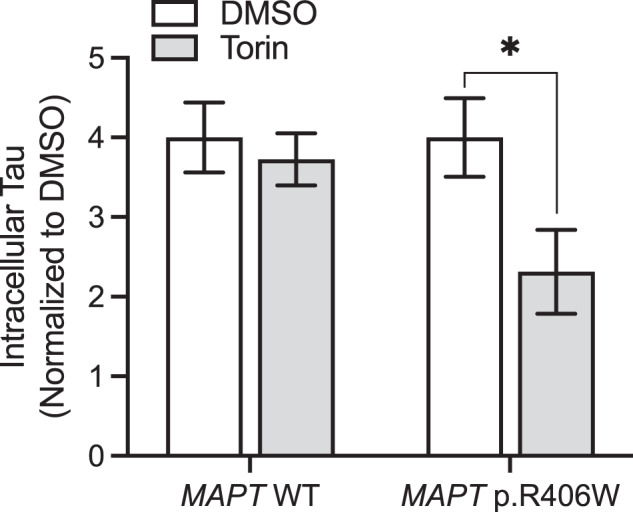
Torin-1 treatment reduces tau specifically in *MAPT* p.R406W neurons. iPSC-derived neurons from *MAPT* p.R406W and isogenic, controls were cultured for 6 weeks prior to treatment with torin-1 or DMSO, control for 5 h. Tau was measured in cells lysates using IP/MS. Graphs represent mean ± SEM. Significance was determined using an unpaired, *t*-test. *p < 0.05.

Tau is degraded by lysosomal pathways [10, 63, 64], and here, we show that *MAPT* p.R406W neurons and 3D cerebral organoids exhibit disrupted lysosomal function. Thus, we asked whether pharmacologically targeting protein degradation pathways would enhance tau degradation. mTOR is the catalytic subunit of mTORC1 and mTORC2, which plays a role in lysosomal biogenesis, activity, and positioning. *MAPT* p.R406W and isogenic, control neurons were treated with a dual mTORC1 and mTORC2 inhibitor, torin-1, or DMSO control, and cell lysates were analyzed by IP/MS. Torin-1 treatment led to a significant reduction in intracellular tau in *MAPT* p.R406W neurons without altering tau levels in control neurons (Fig. 8).

## Discussion

The *MAPT* p.R406W mutation causes autosomal dominant FTLD-tau. However, the pathogenic events triggered by the expression of *MAPT* p.R406W are not fully understood. Current cellular and animal models used to study FTLD-tau and other tauopathies have several limitations. Most cellular and transgenic animal models rely on overexpression of a mutant transgene comprising a single tau isoform, which may produce effects that are a function of protein overexpression and possible off-target effects rather than a disease-relevant phenotype. Thus, our understanding of how tau is metabolized in the human brain has been obtained from experimental paradigms that do not fully capture physiological conditions relevant to human tauopathies. Here, we show that lysosomal biogenesis, autophagosome machinery, and downstream protein degradation pathways are disrupted in FTLD-tau brains expressing the *MAPT* p.R406W mutation and that the *MAPT* p.R406W is sufficient to induce these changes in human neurons and cerebral organoids, leading to altered lysosomal function. Finally, we show that treatment with mTORC1 and mTORC2 inhibitors reduce tau levels specifically in *MAPT* p.R406W neurons.

FTLD-tau caused by *MAPT* p.R406W results in an overall defect in lysosomal machinery. TFEB, which is the master regulator of lysosomal function, was found to be elevated in the FTLD-tau brain tissue homogenates [46]. This increase could reflect changes in neurons and glia. Similarly, we found that *TFEB* transcript levels were elevated in the *MAPT* p.R406W brain tissue. TFEB promotes expression of lysosomal genes and autophagosome formation [37, 49, 65], and many of these genes are also differentially expressed in *MAPT* p.R406W brains. Modulation of lysosomal pathways in mouse models of tauopathy modifies tau clearance and pathology [11–13]. It has been shown that overexpression of TFEB in mouse models of tauopathy (PS19 and rTg4510) activates the lysosomal pathways, leading to reduced tau aggregation and lipofuscin granules and restored synaptic function [12, 13]. TFEB has also been shown to play an essential role in mediating lysosomal-mediated exocytosis and spreading of tau [66]. Additionally, reduced expression of *ATG* genes suggest that autophagy is compromised in *MAPT* p.R406W brain tissue. ATG5*-*ATG12*-*ATG16L1 complex is required at the upstream stages of autophagosomes formation. ATG2a and ATG2b are involved in recruiting additional ATG proteins at the p62‐positive autophagosome‐formation site [50, 67, 68]. Thus, multiple stages of autophagosome formation may be disrupted in FTLD-tau brains.

Tau is degraded through several pathways, including the ubiquitin/proteasome pathway and the lysosome pathway [8, 9]. High molecular weight ubiquitinated proteins were elevated in the *MAPT* p.R406W neurons. This phenotype has been reported in other stem cell models of FTLD-tau and suggests that the cells are undergoing cellular and proteostatic stress [24]. Interestingly, mutant tau protein is unable to efficiently activate the lysosome pathway [10]. Our current findings show that *MAPT* p.R406W is sufficient to lead to enhanced co-localization of total and phosphorylated forms of tau with LAMP1-positive degradative vesicles. This could be due the inefficient activation of the lysosome pathway, reduced degradative capacity within the vesicles, or a feedback loop in which both occur. Consistent with prior reports [36], we do not observe bona-fide aggregated material in *MAPT* p.R406W neurons. With aging, the observed build-up of tau in dysfunctional lysosomes could lead to neurofibrillary tangles.

Efficient bulk cargo degradation and recycling is critical for the homeostasis of the polarized neurons and occurs in the cell body [54]. Endosomes and immature lysosomes travel along the neurites and fuse with the active, LAMP1-positive, lysosomes in the soma [69]. LAMP1-vesicles were distributed away from the nuclear membrane towards the neurites in *MAPT* p.R406W neurons, and this defect was reversed upon correction of the mutant allele. Interestingly, similar phenotypes have been reported in cell lines from Parkinson’s disease patients [70]. LAMP1-vesicle distribution could be due to defects in vesicle trafficking; however, the absence of changes in early endosome distribution as well as the altered lysosomal size suggests there are specific functional defects in the lysosome.

Lysosomes represent a heterogeneous population of vesicles that differ in size, function, and maturity. In addition to a reduction in acidity, we observed an increase in lysosomal enzymes and proteolytic activity in mutant neurons, which is consistent with a phenotype described in lysosomal storage disorders. Secondary elevation of lysosomal enzyme activity, when evaluated in cell lysates under the correct buffer conditions, is a compensatory mechanism found in cells from patients and cell models of lysosomal storage diseases [71, 72]. Loss-of-function mutations in lysosomal genes lead to the accumulation of undegraded material that changes the pH and environment in the lysosomal affecting its degradative capacity [72–74]. *MAPT* p.R406W neurons exhibit an increase in active Cathepsin D along with increased DQ-BSA staining, pointing to more proteolytic activity in the cell. However, the observed elevated proteolytic activity occurs in an environment with enlarged LAMP1-positive vesicles, reduced acidity, and an increase in β-glucuronidase. Together, these data suggest that a number of proteolytic enzymes are building up in vesicles that lack degradative capacity. Phenotypes consistent with those observed in lysosomal storage diseases are increasingly reported in neurodegenerative disease [70, 75–81].

In conclusion, we used iPSC-derived neurons and cerebral organoids to demonstrate that lysosomal dysfunction in FTLD-tau brains may be triggered by the *MAPT* mutation. Targeting these defects by increasing the lysosomal-mediated degradation will likely be effective in treating multiple tauopathies [77].

## Supplementary information

Supplemental Figure Legends

Supplemental Figure 1

Supplemental Figure 2

Supplemental Figure 3

Supplemental Figure 4

Supplemental Figure 5

Supplemental Figure 6

Supplemental Tables

## Data Availability

The datasets analyzed during the current study are available in the Synpase repository: https://www.synapse.org/#!Synapse:syn12181323.
