# Supplementary material for: Defective proteostasis in induced pluripotent stem cell models of frontotemporal lobar degeneration
Source: Transl Psychiatry. 2022 Dec 10;12:508. doi: 10.1038/s41398-022-02274-5 (PMC9734180; doi:10.1038/s41398-022-02274-5)
Supplement: Supplementary file 1 — Supplemental Figure Legends [file 41398_2022_2274_MOESM1_ESM.docx]

**Supplemental Figure Legends**

**Supplementary Figure 1: Characterization of patient-derived and isogenic iPSC.** Representative images of *MAPT* p.R406W (A, F11362.1 and C, F11421.12) and CRISPR/Cas9-edited, isogenic control (B, F11362.1Δ1B06 and D, F11421.12Δ2A07) iPSCs. Sanger sequencing. Karyotyping. Immunostaining for pluripotency markers. Spontaneous differentiation of patient lines (A, C) into cells within the three germ layers evaluated by qPCR. Graph represents mean ± SEM.

**Supplemental Figure 2: Human iPSC-neurons expressing the *MAPT* p.R406W mutation exhibit increase in total tau and phospho-tau.** A. Immunoblots of cell lysates (10μg total protein) were probed with AT180 (ptau-Thr231), total tau, pTFEB (Ser112), TFEB and Rab7 antibodies. B-F. Quantification of protein analyte levels in the *MAPT* p.R406W neurons and isogenic controls. Graph represents mean ± SEM. Significance was determined using an unpaired, t-test. *, p<0.05.

**Supplementary Figure 3: LAMP1-positive staining in Tuj1-positive neurons.** Human iPSC-neurons neurons from a *MAPT* p.R406W mutation carrier and the isogenic, CRISPR-corrected controls (wild-type (WT)) were cultured in cortical maturation media for 6 weeks. A. Representative immunostaining for LAMP1 (red), Tuj1 (green) and DAPI (blue) illustrates LAMP1-positive vesicles in Tuj1-positive cells. LAMP1 quantification shown in Figure 1. Scale bar, 10 microns.

**Supplemental Figure 4: Human iPSC-neurons expressing the *MAPT* p.R406W mutation display defects in lysosomal morphology in an independent donor cell line.** Human iPSC-neurons neurons from a *MAPT* p.R406W mutation carrier (F11421.12) and the isogenic, CRISPR/Cas9-corrected control (wild-type (WT)) were differentiated into cortical neurons and were cultured for 6 weeks prior to analysis. A. Representative immunostaining for LAMP1 (red) and DAPI (blue) illustrates altered LAMP1-positive vesicles in the *MAPT* p.R406W neurons compared with isogenic controls. Scale bar, 10 microns. White arrow showing LAMP1-positive vesicles in the neurites of *MAPT* p.R406W neurons. B. Distance of LAMP1-positive vesicles from the nuclear membrane is significantly greater in *MAPT* p.R406W neurons (n=54 cells) compared to isogenic controls (n=73 cells). C. The size of LAMP1-positive vesicles in the soma is significantly larger in *MAPT* p.R406W neurons (n=46 cells) compared with the isogenic controls (n= 69 cells). E. The number of LAMP1-positive vesicles within the soma is significantly reduced in *MAPT* p.R406W neurons (n=10 cells) compared to isogenic controls (n= 10 cells). All quantification was performed in Tuj1-positive cells. Graphs represent mean ± SEM. Significance was determined using an unpaired, t-test. *, p<0.05; ***, p<0.001; ****, p<0.0001.

**Supplemental Figure 5: Early endosome morphology is unchanged human iPSC-neurons expressing the *MAPT* p.R406W mutation.** Human iPSC-neurons neurons from a *MAPT* p.R406W mutation carrier and the isogenic, CRISPR/Cas9-corrected control (wild type (WT)) were cultured in cortical maturation media for 6 weeks. A. Representative immunostaining for EEA1 (red) and DAPI (blue) illustrates similar patterns of EEA1-positive staining in *MAPT* p.R406W neurons compared with isogenic control neurons. Scale bar, 10 microns. B. Quantification of distance of EEA1 from the nuclear membrane illustrates no significant difference between *MAPT* p.R406W neurons (n=35 cells) compared to controls (n=36 cells). C. Quantification of EEA1 vesicle size illustrates no significant difference between in *MAPT* p.R406W mutation carrier neurons (n=4 cells) compared to controls (n=4 cells). Graphs represent mean ± SEM. All comparisons were not significant, as determined by an unpaired, t-test.

**Supplemental Figure 6: Human iPSC-neurons expressing the *MAPT* p.R406W mutation exhibit defects in lysosomal function in an independent donor cell line.** Human iPSC-neurons neurons from a *MAPT* p.R406W mutation carrier (F11421.12) and isogenic, CRISPR/Cas9-corrected control (wild-type (WT)) were differentiated into cortical neurons and were cultured for 6 weeks prior to analysis. A. *MAPT* p.R406W neurons exhibit reduced LysoTracker staining. Live cells were incubated with LysoTracker and were imaged as described in Methods. Representative images of LysoTracker-stained neurons are shown in gray scale for clarity. Scale bar, 10 microns. Lower panel represents magnification of the cells in the black box. B. Quantification of the intensity of LysoTracker staining in soma of *MAPT* p.R406W neurons (n=78 cells) and isogenic controls (n=70 cells). C. *MAPT* p.R406W neurons exhibit increased DQ-BSA staining. Live cells were incubated with DQ-BSA and were imaged as described in Methods. Representative images of DQ-BSA-stained neurons are shown in gray scale for clarity. Scale bar, 10 microns. Lower panel represents magnification of the cells in the black box. D. Quantification of the intensity of DQ-BSA staining in soma of *MAPT* p.R406W neurons (n=91 cells) and isogenic controls (n=94 cells). Graphs represent mean ± SEM. Significance was determined using an unpaired, t-test. ****, p<0.0001.
