# Supplementary figures and images for: Defective proteostasis in induced pluripotent stem cell models of frontotemporal lobar degeneration
Source: Transl Psychiatry. 2022 Dec 10;12:508. doi: 10.1038/s41398-022-02274-5 (PMC9734180; doi:10.1038/s41398-022-02274-5)

# Supplemental Figure 1

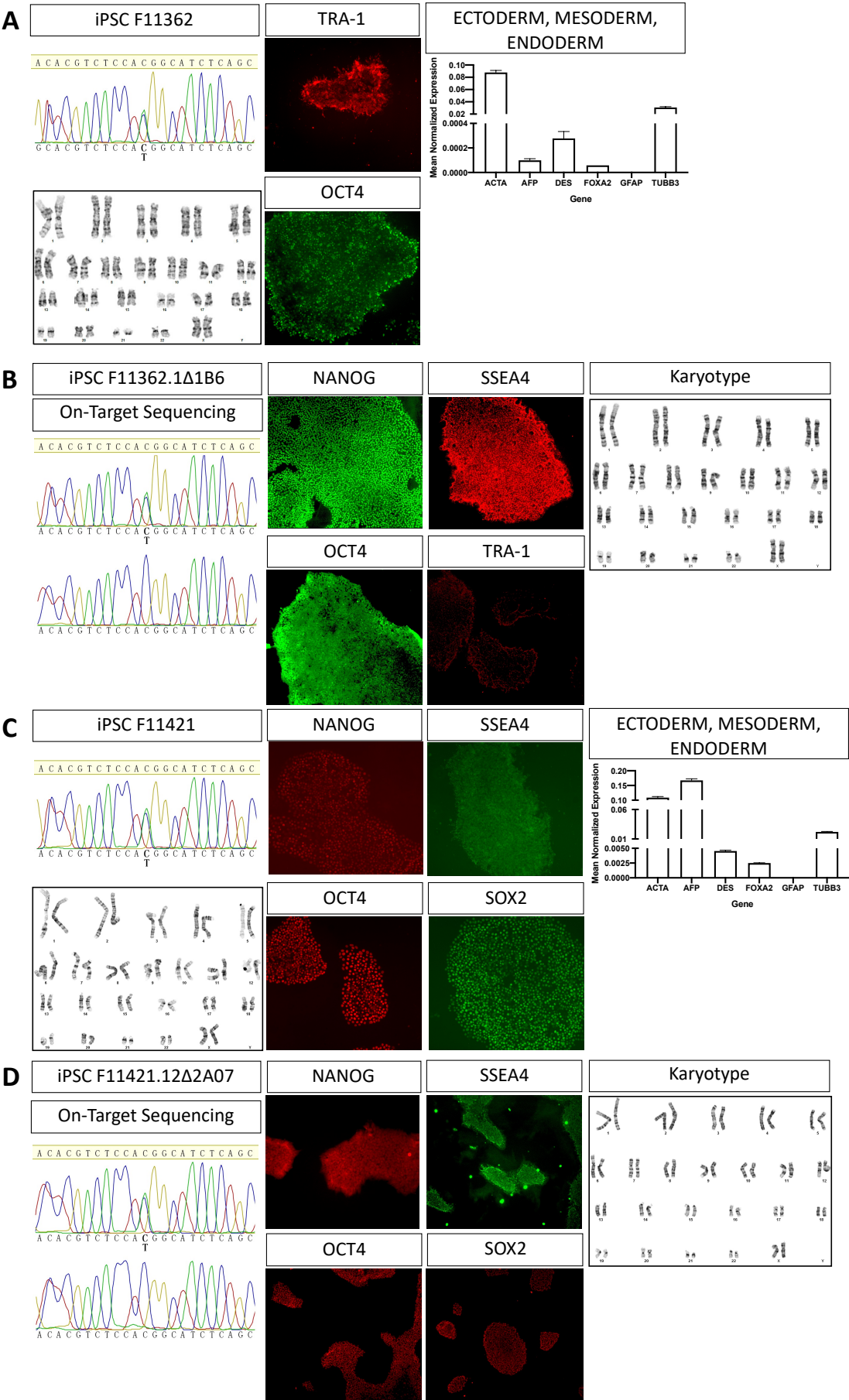

Supplement: Supplementary file 2 — Supplemental Figure 1 [file 41398_2022_2274_MOESM2_ESM.pdf]

Supplemental Figure 2

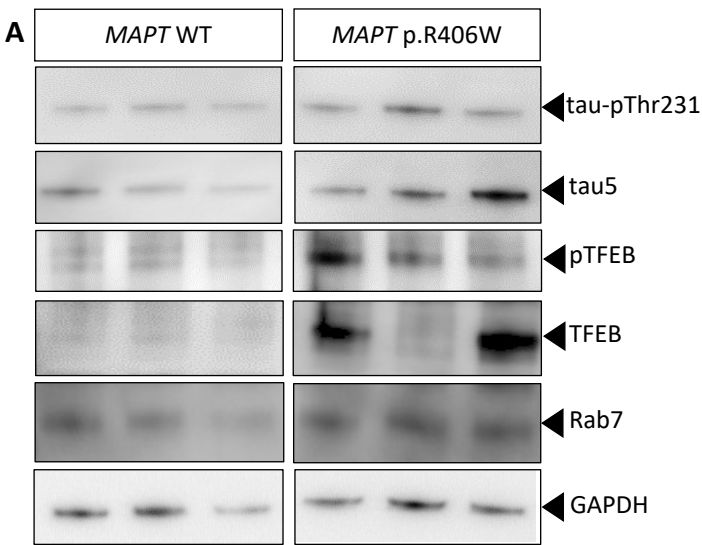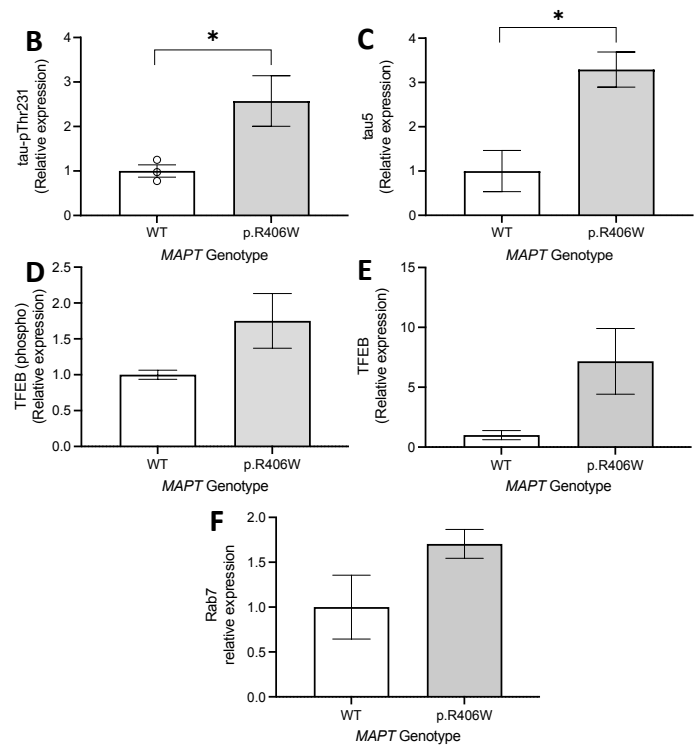

Supplement: Supplementary file 3 — Supplemental Figure 2 [file 41398_2022_2274_MOESM3_ESM.pdf]

Supplemental Figure 3

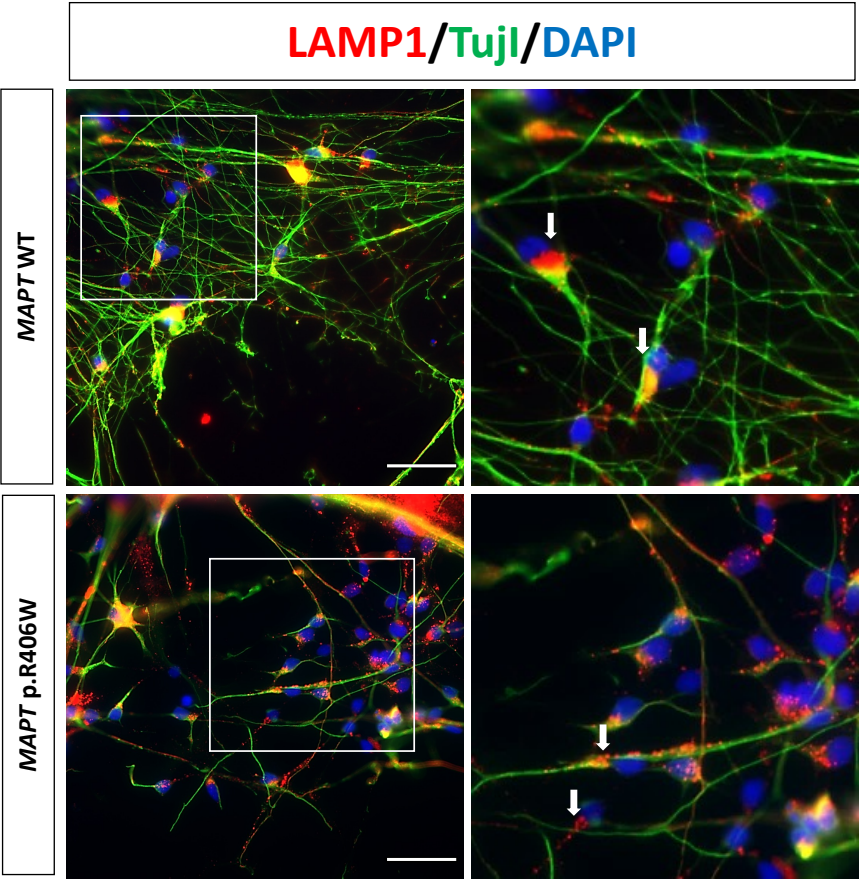

Supplement: Supplementary file 4 — Supplemental Figure 3 [file 41398_2022_2274_MOESM4_ESM.pdf]

# Supplemental Figure 4

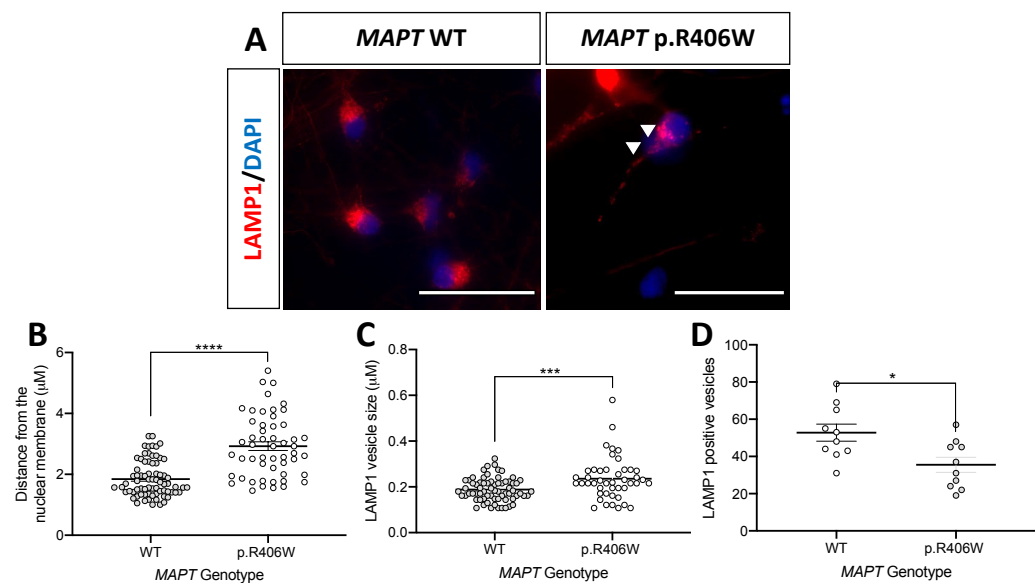

Supplement: Supplementary file 5 — Supplemental Figure 4 [file 41398_2022_2274_MOESM5_ESM.pdf]

Supplemental Figure 5

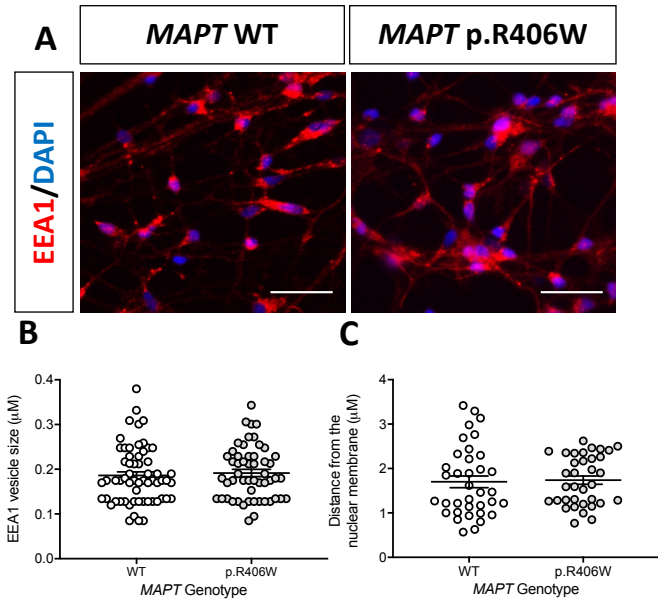

Supplement: Supplementary file 6 — Supplemental Figure 5 [file 41398_2022_2274_MOESM6_ESM.pdf]

Supplemental Figure 6

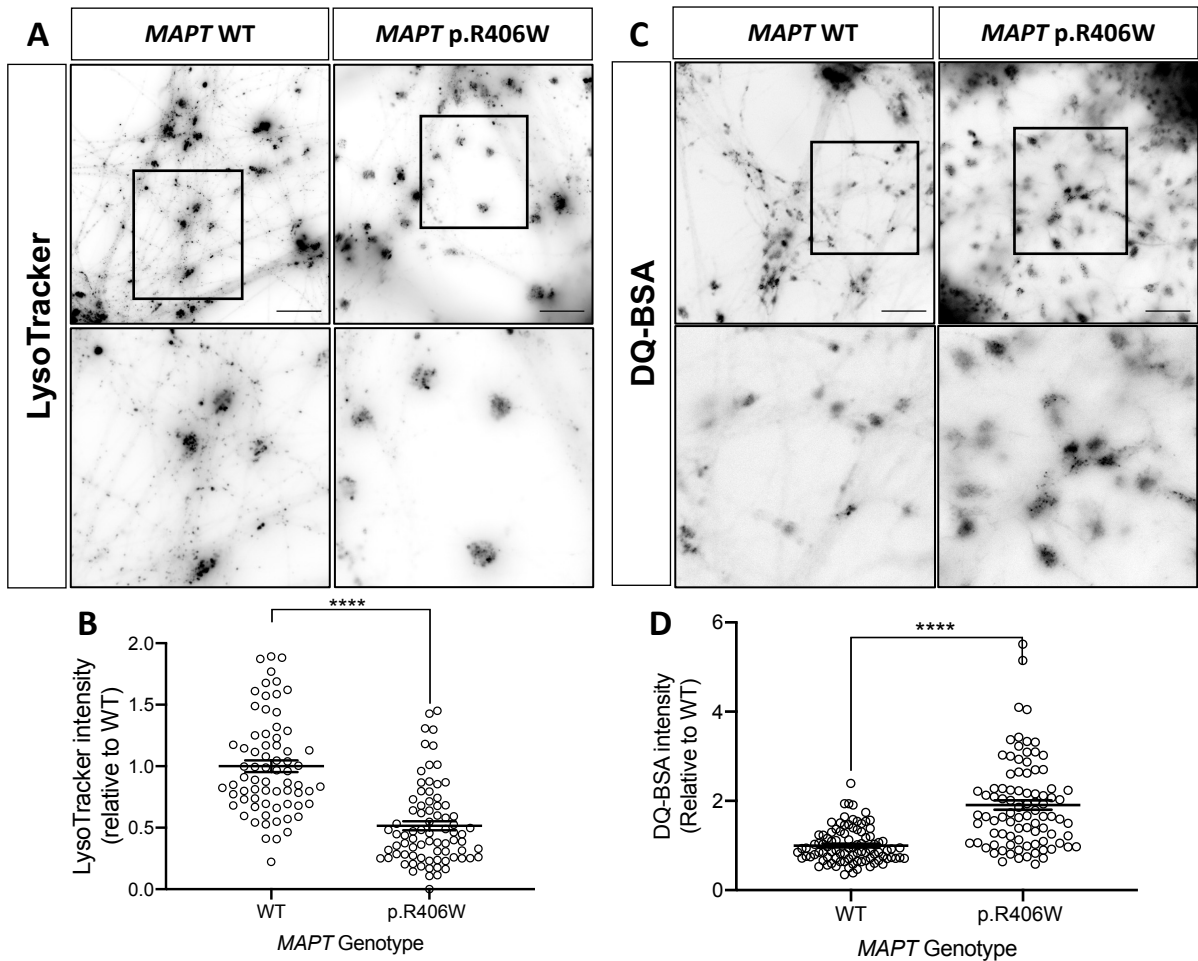

Supplement: Supplementary file 7 — Supplemental Figure 6 [file 41398_2022_2274_MOESM7_ESM.pdf]
